# The impact of visual dysfunctions in recent-onset psychosis and clinical high-risk state for psychosis

**DOI:** 10.1038/s41386-022-01385-3

**Published:** 2022-08-18

**Authors:** Johanna M. Schwarzer, Inga Meyhoefer, Linda A. Antonucci, Lana Kambeitz-Ilankovic, Marian Surmann, Olga Bienek, Georg Romer, Udo Dannlowski, Tim Hahn, Alexandra Korda, Dominic B. Dwyer, Anne Ruef, Shalaila S. Haas, Marlene Rosen, Theresa Lichtenstein, Stephan Ruhrmann, Joseph Kambeitz, Raimo K. R. Salokangas, Christos Pantelis, Frauke Schultze-Lutter, Eva Meisenzahl, Paolo Brambilla, Alessandro Bertolino, Stefan Borgwardt, Rachel Upthegrove, Nikolaos Koutsouleris, Rebekka Lencer, the PRONIA Consortium, Alkomiet Hasan, Claudius Hoff, Ifrah Khanyaree, Aylin Melo, Susanna Muckenhuber-Sternbauer, Yanis Köhler, Ömer Öztürk, Nora Penzel, David Popovic, Adrian Rangnick, Sebastian von Saldern, Rachele Sanfelici, Moritz Spangemacher, Ana Tupac, Maria Fernanda Urquijo, Johanna Weiske, Antonia Wosgien, Camilla Krämer, Karsten Blume, Dominika Julkowski, Nathalie Kaden, Ruth Milz, Alexandra Nikolaides, Mauro Silke Vent, Martina Wassen, Christina Andreou, Laura Egloff, Fabienne Harrisberger, Ulrike Heitz, Claudia Lenz, Letizia Leanza, Amatya Mackintosh, Renata Smieskova, Erich Studerus, Anna Walter, Sonja Widmayer, Chris Day, Sian Lowri Griffiths, Mariam Iqbal, Mirabel Pelton, Pavan Mallikarjun, Alexandra Stainton, Ashleigh Lin, Paris Lalousis, Alexander Denissoff, Anu Ellilä, Tiina From, Markus Heinimaa, Tuula Ilonen, Päivi Jalo, Heikki Laurikainen, Antti Luutonen, Akseli Mäkela, Janina Paju, Henri Pesonen, Reetta-Liina Säilä, Anna Toivonen, Otto Turtonen, Sonja Botterweck, Norman Kluthausen, Gerald Antoch, Julian Caspers, Hans-Jörg Wittsack, Ana Beatriz Solana, Manuela Abraham, Timo Schirmer, Carlo Altamura, Marika Belleri, Francesca Bottinelli, Adele Ferro, Marta Re, Emiliano Monzani, Maurizio Sberna, Armando D’Agostino, Lorenzo Del Fabro, Giampaolo Perna, Maria Nobile, Alessandra Alciati, Matteo Balestrieri, Carolina Bonivento, Giuseppe Cabras, Franco Fabbro, Marco Garzitto, Sara Piccin

**Affiliations:** 1 Institute for Translational Psychiatry, University of Muenster, Muenster, Germany; 2 Department of Basic Medical Science, Neuroscience and Sense Organs, University of Bari Aldo Moro, Bari, Italy; 3 Department of Education, Psychology, Communication, University of Bari Aldo Moro, Bari, Italy; 4 Department of Psychiatry and Psychotherapy, Ludwig Maximilian University Munich, Munich, Germany; 5 Department of Psychiatry and Psychotherapy, Faculty of Medicine and University Hospital, University of Cologne, Cologne, Germany; 6 Department of Child Adolescence Psychiatry and Psychotherapy, University of Muenster, Muenster, Germany; 7 Department of Psychiatry and Psychotherapy, University of Luebeck, Luebeck, Germany; 8 Department of Psychiatry, Icahn School of Medicine at Mount Sinai, New York, NY USA; 9 Department of Psychiatry, University of Turku, Turku, Finland; 10 Melbourne Neuropsychiatry Centre, University of Melbourne and Melbourne Health, Melbourne, VIC Australia; 11 Department of Psychiatry and Psychotherapy, Medical Faculty, Heinrich-Heine University, Duesseldorf, Germany; 12 University Hospital of Child and Adolescent Psychiatry and Psychotherapy, University of Bern, Bern, Switzerland; 13 Department of Psychology, Faculty of Psychology, Airlangga University, Surabaya, Indonesia; 14 Department of Neurosciences and Mental Health, Fondazione IRCCS Ca’ Granda Ospedale Maggiore Policlinico, Milan, Italy; 15 Department of Pathophysiology and Transplantation, University of Milan, Milan, Italy; 16 Department of Psychiatry, Psychiatric University Hospital, University of Basel, Basel, Switzerland; 17 Institute for Mental Health, and Centre for Human Brain Health, School of Psychology, University of Birmingham, Birmingham, UK; 18 Birmingham Early Intervention Service, Birmingham Womens and Childrens NHS Foundation Trust, Birmingham, UK; 19 Institute of Psychiatry, Psychology and Neuroscience, King’s College London, London, UK; 20 Max-Planck-Institute of Psychiatry, Munich, Germany; 21 General Electric Global Research Inc, Niskayuna, NY USA; 22 Programma 2000, Niguarda Hospital, Milan, Italy; 23 San Paolo Hospital, Milan, Italy; 24 Villa San Benedetto Menni, Albese con Cassano, CO Italy; 25 Department of Medical Area, University of Udine, Udine, Italy; 26 IRCCS Scientific Institute “E. Medea”, Polo FVG, Udine, Italy

**Keywords:** Predictive markers, Psychosis

## Abstract

Subtle subjective visual dysfunctions (VisDys) are reported by about 50% of patients with schizophrenia and are suggested to predict psychosis states. Deeper insight into VisDys, particularly in early psychosis states, could foster the understanding of basic disease mechanisms mediating susceptibility to psychosis, and thereby inform preventive interventions. We systematically investigated the relationship between VisDys and core clinical measures across three early phase psychiatric conditions. Second, we used a novel multivariate pattern analysis approach to predict VisDys by resting-state functional connectivity within relevant brain systems. VisDys assessed with the Schizophrenia Proneness Instrument (SPI-A), clinical measures, and resting-state fMRI data were examined in recent-onset psychosis (ROP, *n* = 147), clinical high-risk states of psychosis (CHR, *n* = 143), recent-onset depression (ROD, *n* = 151), and healthy controls (HC, *n* = 280). Our multivariate pattern analysis approach used pairwise functional connectivity within occipital (ON) and frontoparietal (FPN) networks implicated in visual information processing to predict VisDys. VisDys were reported more often in ROP (50.34%), and CHR (55.94%) than in ROD (16.56%), and HC (4.28%). Higher severity of VisDys was associated with less functional remission in both CHR and ROP, and, in CHR specifically, lower quality of life (Qol), higher depressiveness, and more severe impairment of visuospatial constructability. ON functional connectivity predicted presence of VisDys in ROP (balanced accuracy 60.17%, *p* = 0.0001) and CHR (67.38%, *p* = 0.029), while in the combined ROP + CHR sample VisDys were predicted by FPN (61.11%, *p* = 0.006). These large-sample study findings suggest that VisDys are clinically highly relevant not only in ROP but especially in CHR, being closely related to aspects of functional outcome, depressiveness, and Qol. Findings from multivariate pattern analysis support a model of functional integrity within ON and FPN driving the VisDys phenomenon and being implicated in core disease mechanisms of early psychosis states.

## Introduction

**Fig. 1:**
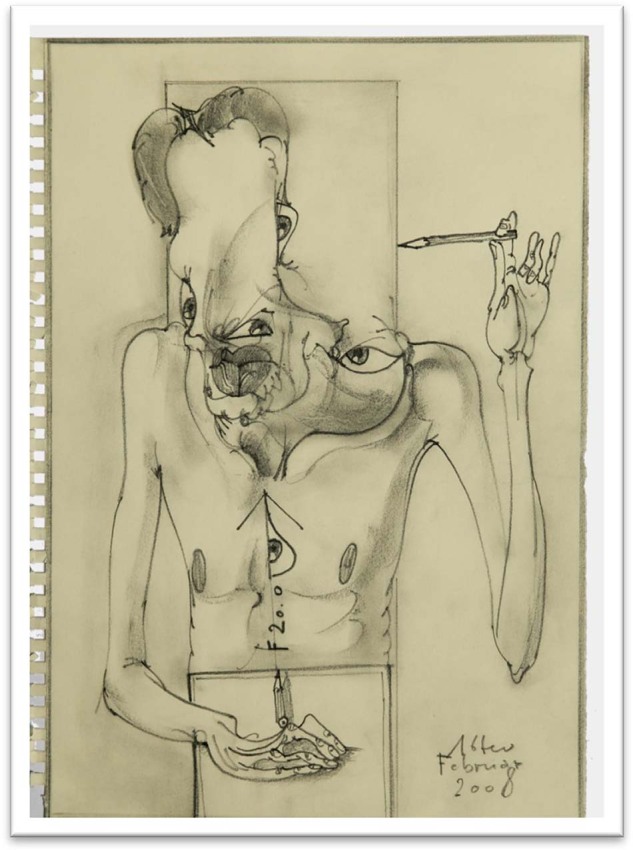
Prominent example of visual distortions illustrated by a patient with a psychotic disorder. Copyright courtesy to the artist.

Perceptual deficits in terms of hallucinations are diagnostically indicative for psychotic disorders such as schizophrenia [1] but may also occur at more subtle levels [2]. Recent research emphasized dysfunctions within the visual system, specifically early visual processing impairments within the dorsal visual stream [3, 4], to be an important subject to study. About 50–60% of patients diagnosed with schizophrenia report visual dysfunctions (VisDys) affecting brightness, motion, form, color perception or distorted perception of one’s face (Fig. 1) [5, 6], in contrast to patients with non-psychotic disorders [7, 8]. These subtle VisDys are often underrecognized during clinical examination despite their clinical relevance related to suicidal ideation, cognitive impairment, or poorer treatment response [5]. Studying VisDys and their neurobiological underpinnings could foster our understanding of basic disease mechanisms implicated in psychotic disorders [5] as impairments of visual processing, e.g., decreased contrast sensitivity [9, 10], disturbed forward/backward masking [11], decreased visual context surround suppression [12], or general altered perceptual organization in schizophrenia [13] have been reported from psychophysiological studies. Such dysfunctions may be explained by deficient optimization of response levels and other deficits of multiple visual integration along visual processing pathways in the brain [3, 14, 15].

Most studies about VisDys in schizophrenia included patients at a stable, chronic state of the illness. Furthermore, VisDys are also considered so-called basic symptoms being present even years before the diagnosis of a psychotic disorder [7, 8, 16]. High-risk patients, who report VisDys, may be even more sensitive to convert to psychosis than those without VisDys [17]. This suggests deeper insights into VisDys and their dynamic in early states of psychosis could hold beneficial information for clinical practice, and especially, on neurobiologically determined brain dysfunction mediating susceptibility to psychosis. However, only little research has focused on the neurobiological underpinnings of VisDys specifically in early states of psychosis and/or in comparison to other disorders, particularly depression [8, 16].

The Personalised Prognostic Indicators for early Psychosis management (PRONIA, https://www.pronia.eu) consortium offers the unique chance to systematically study, first, the psychophysiological phenomenon of VisDys in a large sample of adolescents and young adults comprising three diagnosis groups: recent-onset psychosis (ROP), clinical high-risk state of psychosis (CHR) and recent-onset depression (ROD). VisDys in daily life were here assessed using the Schizophrenia Proneness Instrument-Adult Scale (SPI-A) [6], an extensively validated and used scale to assess basic symptoms indicating increased risk to psychosis [8, 18–21]. Second, resting-state imaging data on intrinsic brain networks were also assessed in the PRONIA sample and analyzed based on work by Dosenbach et al. [22]. This yielded 12,720 functional connectivities between 160 regions of interest (ROIs) across the whole brain comprising six subnetworks (cerebellum, cingulo-opercular, default, frontoparietal, sensorimotor and occipital). Regarding our interest in primary networks for visual information processing, especially the dorsal visual stream, we focused on two subnetworks, namely the occipital (ON) and frontoparietal (FPN) intrinsic networks. The ON was chosen for comprising primary visual processing pathways while the FPN is widely suggested to modulate attention related to visual information processing at higher cognitive levels [23, 24].

Provided the large PRONIA sample [25], a multivariate pattern analysis approach was chosen to study the complex relationships between ON, FPN, and VisDys. This approach enables the consideration of multiple interactions within brain systems as required for the study of inherently heterogeneous collectives of mental disorders beyond clinical evaluation [26]. In addition, this approach offers the opportunity to investigate the predictive value of these brain networks for the classification of VisDys presence or absence on an individual level.

Three major research questions drove our analyses. First, are VisDys specific to the psychosis spectrum at early stages of a mental disorder? Second, are VisDys associated with clinical characteristics, i.e., higher symptom load and worse functional outcome? Third, is functional intrinsic connectivity within ON and FPN related to the presence or absence of VisDys, and if so, are there differences in this relationship across the psychosis spectrum?

## Methods

### Sample characteristics

A total sample of 721 participants was drawn from the PRONIA database [27], including 147 with ROP, 143 participants at CHR, 151 with ROD, and 280 HC. Participants were recruited through early detection units across seven European university sites [27]. Written informed consent was obtained from all participants and from legal guardians for underage participants, respectively. Each site received ethical approval from their referring ethics committee conforming with the Helsinki Declaration [27]. General inclusion criteria were age between 15 and 40 years, sufficient language skills for participation, and sufficient capacity to consent. General exclusion criteria included an IQ below 70, current or past neurological or somatic disorder of the brain including head trauma with loss of consciousness (>5 min); any medical contraindications for MRI; current or past (past 6 months) alcohol or substance dependency.

Additionally, ROP participants had to meet the DSM-IV-TR criteria for an affective or non-affective psychotic episode within the last 3 months, with the start of psychotic symptoms within the 24 months preceding screening date. Exclusion criteria for ROP participants were any antipsychotic medication for longer than 90 days (within the past 24 months) with a daily dose rate at or above the minimum dosage of DGPPN S3-guidelines [28]. Participants were excluded if psychotic symptoms were drug-induced and, therefore, abstinence of any drugs, including cannabis and alcohol, for at least 1 month, was mandatory.

A CHR state was alternatively defined by the basic symptom criterion “Cognitive Disturbances” [6, 16, 18] and any of the adapted PRONIA ultra-high-risk criteria [27]. It was carefully checked through the questionnaire and in single case consensus conferences that diagnosis-defining criteria were not induced by drug use. Exclusion criteria specific to the CHR group included antipsychotic medication for more than 30 days and intake of antipsychotic medication within the past 3 months before baseline assessments at or above a minimum dosage of first-episode psychosis according to DGPPN S3-guideline [28].

ROD participants had to meet the DSM-IV-TR Major Depressive Episode criteria within their lifetime and major depressive disorder (MDD) criteria within the past 3 months, while the duration of the first depressive episode must not exceed 24 months. Specific exclusion criteria for ROD participants were more than one MDD episode during the lifetime and the intake of antipsychotic medication analogous to the CHR exclusion criteria.

Exclusion criteria specific to HC participants included current or past DSM-IV-TR-Axis-I disorder; history of CHR criteria; affective or non-affective psychosis or major affective disorder of first-degree relatives, and intake of psychopharmacological substances.

### Clinical assessments

**Fig. 2:**
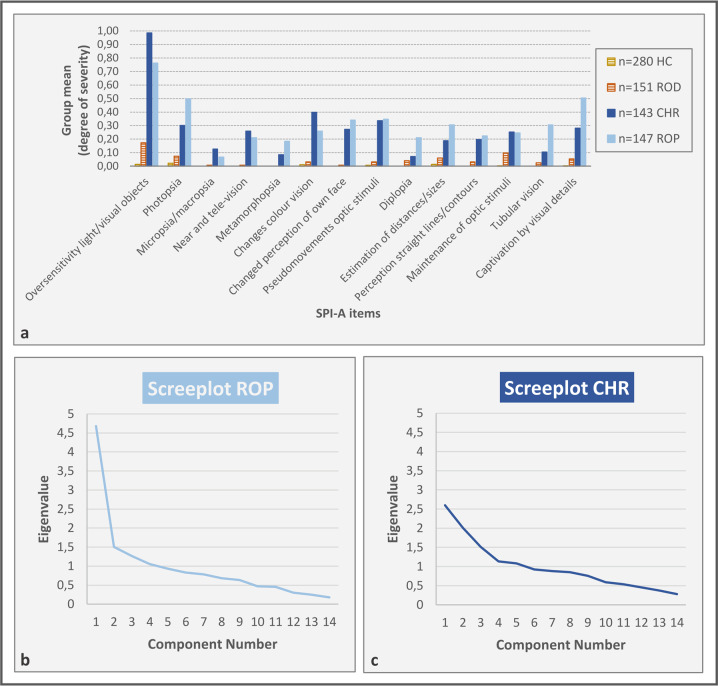
Summary of VisDys item means and underlying component structure by group. Displays degrees of severity for each of the 14 VisDys items by group derived from SPI-A (**a**) and visualization of the number of components underlying VisDys sum score for ROP and CHR groups (scree plots from PCA analyses in **b**, **c**, respectively). For more details see Tables S2 and S3.

Fourteen items from the SPI-A were selected to represent different aspects of VisDys [6] (Fig. 2a and Table S1). Participants reported the severity of the respective VisDys defined by the maximum frequency within the past 3 months (0 = never, 6 = daily). A sum score was computed over the 14 SPI-A items for each participant individually as reported previously [29]. Additionally, participants were categorized into two groups based on this sum score, i.e., VisDys^+^ (sum score > 0) and VisDys^−^ (sum score = 0), respectively, for further analyses.

Other clinical assessments included the Beck Depression Inventory-II (BDI-II) [30], the Positive and Negative Syndrome Scale (PANSS) [31], the Functional Remission in General Schizophrenia (FROGS) [32] scale (including daily life, social functioning, treatment subscales), the GF Role [33] and GF Social [34] for global role and social functioning, and the World Health Organization-Quality of Life (QoL) scale that includes the subscales physical, psychological, social relationships, and environment [35].

Additionally, the Rey-Osterrieth Complex Figure Test whole score (ROCF) [36, 37], as a neuropsychological measurement of visuospatial constructability was assessed to study whether VisDys may be associated with the ability to see an object or picture as a set of parts and then construct a replica of the original from these parts [38]. This was tested immediately (ROCF immediate) and 30 min (ROCF delayed) after the object presentation.

### Statistical analyses of clinical data

Demographic characteristics, behavioral data, and VisDys parameters were compared between groups using one-way ANOVAs, *X*^2^*-*tests, and crosstabs. Principal component analysis (PCA) with orthogonal rotation (varimax) was applied on SPI-A items in groups separately. VisDys sum scores were correlated with clinical measures using nonparametric correlations with Kendall’s Tau (*τ*).

Depending on the number of group comparisons (HC-ROD, HC-CHR, HC-ROP, ROD-CHR, ROD-ROP, CHR-ROP resulting in six comparisons) and subscales of each measurement we corrected for multiple testing using Bonferroni–Holm-corrected alpha levels [39], corrected *p* values are reported throughout.

### Assessment and analyses of resting-state activity

To facilitate the evaluation of real-world generalizability, a minimal MRI harmonization protocol was implemented across all PRONIA-sites. While acquiring brain resting-state activity, subjects were instructed to keep their eyes open and not to think about anything. For details including preprocessing and analyses of resting-state MRI see Supplementary Material and ref. [40].

Based on our a priori hypothesis, we focused on ROIs associated with intrinsic brain networks as defined by the Dosenbach functional atlas [22] which are involved in primary visual processing along the dorsal visual stream. This resulted in ON (*N* = 22 ROIs) and FPN (*N* = 21 ROIs, Table S4), comprising 231 pairwise connectivities for ON, 210 connectivities for FPN, and 903 connectivities for combined ON-FPN (Tables S3, S9–S12).

### Machine-learning analyses

A total of 135 ROP, 128 CHR, and 134 ROD participants, for whom rsfMRI was available, were entered in the pipeline. Since prevalence of VisDys in HC was very low, HC were excluded from this approach.

For multivariate pattern analysis we used the NeuroMiner software (version 1.0; www.proniapredictors.eu/neurominer/index.html) for the classification of VisDys^−^ vs. VisDys^+^ indicating the absence or presence of VisDys within the past 3 months based on resting-state network connectivities in ON and FPN as defined above. Individual models for each of the groups (ROP, CHR, ROD) were trained and parameters were optimized using a repeated nested leave-one-site-out cross-validation design [41, 42]. Thus, hyperparameter optimization was done within an inner cross-validation cycle (CV1; 6 folds) and, subsequently, the best performing model was applied to an outer CV cycle (CV2; 7 folds/study sites). Thus, in each CV2 cycle one study site was held out to generate geographical generalization [25, 27, 43, 44]. To better identify connections across groups possibly associated with VisDys, we extracted the cross-validation ratio (CVR) for each connectivity in each group for each intrinsic network (for details on CVR and the machine-learning pipeline see Supplementary Material).

## Results

### Clinical characteristics of the sample

Compared to the HC group, ROP, CHR, and ROD scored lower on clinical parameters measuring Qol and Global Functioning (Table 1). Furthermore, all patient groups expressed a higher level of depression, i.e., BDI-II scores, and performed lower on the visuospatial ability task.

**Table 1:** Sociodemographic and clinical characteristics of the original sample (shown are numbers or means with standard deviation (SD), respectively).

	HC	ROD	CHR	ROP	Statistics
*N*^e^	280	151	143	147	
Age (SD)	28.54 (6.43)^c^	29.13 (6.22)^c^	26.97 (4.98)^b^	28.45 (5.53)	*F*(3,717) = 3.57, *p* = 0.014
Sex (m:w)	110:170^d^	71:80^d^	72:71	89:58^a,b^	*X*^2^(3, *N* = 721) = 18.08, *p* = 4.22e−4
VisDys Prevalence (±)	4.28% (12/268)^b,c,d^	16.56% (25/126)^a,c,d^	55.94% (80/63)^a,b^	50.34% (74/73) ^a,b^	*X*^2^(3, *N* = 721) = 185.19, *p* = 6.67e−40
VisDys sum score (SD)	0.08 (0.39)^c,d^	0.64 (2.06)^c,d^	3.85 (5.71)^a,b^	4.46 (8.91)^a,b^	*F*(3,717) = 37.92, *p* = 9.44e−23
WHOQOL physical (SD)	17.64 (1.67)^b,c,d^	12.86 (2.83)^a,d^	13.00 (2.38)^a^	13.63 (2.60)^a,b^	*F*(3,648) = 204.62, *p* = 2.37e−93
WHOQOL psychological (SD)	16.38 (2.05)^b,c,d^	10.47 (2.75)^a,d^	10.59 (3.03)^a,d^	12.14 (3.12)^a,b,c^	*F*(3,654) = 226.32, *p* = 1.11e−100
WHOQOL social (SD)	16.04 (2.65)^b,c,d^	12.33 (3.47)^a^	12.32 (3.62)^a^	12.66 (3.58)^a^	*F*(3,642) = 65.31, *p* = 7.26e−37
WHOQOL environmental (SD)	16.71 (1.84)^b,c,d^	14.45 (2.54)^a^	14.19 (2.24)^a^	13.94 (2.61)^a^	*F*(3,652) = 67.98, *p* = 2.94e−38
FROGS DailyLife (SD)	–	20.80 (4.67)	19.41 (4.43)^d^	20.08 (4.72)^c^	*F*(2,433) = 21.01, *p* = 1.96e−9
FROGS Activities (SD)	–	11.98 (3.16)^d^	8.87 (3.50)^d^	10.32 (3.22)^b,c^	*F*(2,433) = 13.59, *p* = 2.00e−6
FROGS Relationships (SD)	–	20.35 (4.64)^d^	17.03 (4.27)^d^	18.04 (4.77)^b,c^	*F*(2,433) = 19.38, *p* = 8.63e−9
FROGS Health and Treatment (SD)	–	12.58 (2.62)	11.31 (2.51)	11.72 (2.80)	*F*(2,432) = 16.37, *p* = 1.41e−7
PANSS pos (SD)	–	7.66 (1.24)^c,d^	10.17 (3.16)^b,d^	17.51 (6.36)^b,c^	*F*(2,433) = 223.16, *p* = 2.46e−67
PANSS neg (SD)	–	12.27 (4.79)^d^	12.20 (5.77)^d^	16.47 (7.89)^b,c^	*F*(2,433) = 22.01, *p* = 7.90e−10
PANSS gen (SD)	–	26.95 (6.55)^d^	27.74 (7.12)^d^	35.64 (11.01)^b,c^	*F*(2,431) = 46.89, *p* = 2.85e−19
ROCF whole (SD)	34.56 (1.88)^d^	33.98 (2.78)	34.14 (2.71)	33.30 (3.89)^a^	*F*(3,696) = 6.78, *p* = 1.65e−4
ROCF immediate (SD)	24.71 (5.57)^c,d^	23.76 (6.12)^d^	22.84 (6.50)^a,d^	19.57 (7.09)^a,b,c^	*F*(3,696) = 22.15, *p* = 1.05e−13
ROCF delayed (SD)	24.75 (5.31)^c,d^	23.63 (6.15)^d^	22.24 (6.41)^a,d^	19.72 (6.91)^a,b,c^	*F*(3,696) = 23.00, *p* = 3.77e−14
BDI-II (SD)	3.67 (5.24)^b,c,d^	25.85 (13.98)^a,d^	25.75 (12.24)^a,d^	21.91 (12.61)^a,b,c^	*F*(3,659) = 212.69, *p* = 8.61e−98
GF Role (SD)	8.55 (0.75)^b,c,d^	6.24 (1.61)^a,d^	6.18 (1.42)^a,d^	5.19 (1.71)^a,b,c^	*F*(3,712) = 252.89, *p* = 2.97e−114
GF Social (SD)	8.51 (0.82)^b,c,d^	6.46 (1.32)^a,d^	6.47 (1.30)^a,d^	5.63 (1.51)^a,b,c^	*F*(3,712) = 232.52, *p* = 1.91e−107

Indicate significant differences with *p* < 0.05 revealed in post hoc analyses compared to: a = HC; b = ROD; c = CHR; d = ROP, for details see text. Positive and Negative Syndrome Scale: -positive scale (PANSS pos), -negative scale (PANSS neg), -general scale (PANSS gen); Beck Depression Inventory-II (BDI-II); World Health Organization Quality of life: -physical subscale (WHOQOL physical), -psychological subscale (WHOQOL psychological), -social subscale (WHOQOL social), -environmental subscale (WHOQOL environmental); Functional Remission of General Schizophrenia: -daily life subscale (FROGS_DailyLife), -activities subscale (FROGS Activities), -relationships subscale (FROGS Relationships), -health and treatment subscale (FROGS Health and Treatment); Rey-Osterrieth Complex Figure Test: -whole score (ROCF whole), -immediate Score (ROCF immediate), -delayed score (ROCF delayed); Global functioning: -role level (GF Role), -social level (GF Social).

^e^*N* where VisDys data were available.

Comparisons of clinical measures between ROP and CHR, the two groups showing similarly high VisDys prevalence rates and severity (Table 1), indicate ROP expressing higher symptom severity on all PANSS subscales and lower functional remission, i.e., FROGS subscales, and overall functioning, i.e., GF Role and GF Social scales. Regarding visuospatial ability (ROCF), ROP patients performed weaker compared to CHR. On the other hand, ROP participants compared to CHR showed less depressive symptom expression on BDI-II and reported higher levels of Qol (WHOQOL, psychological scale, Table S5). Note, in both groups, higher BDI scores were associated with lower WHOQOL scores, specifically WHOQOL-subscores physical and psychological in CHR and all WHOQOL scores in ROP.

### VisDys characteristics across groups

VisDys prevalence was higher in groups belonging to the psychosis spectrum, i.e., ROP (50.34%) and CHR (55.94%), compared with ROD (16.56%) and HC (4.28%; ROP = CHR > ROD = HC) (Table 1). In line with this, VisDys sum scores in ROP and CHR were also higher than in ROD and HC. Note, VisDys sum scores showed high internal consistency (Cronbachs alpha = 0.78 over all subjects, *n* = 721).

Detailed evaluation of individual SPI-A items by PCA (Fig. 2a) revealed different characteristics across the groups (ROP: KMO (Kaiser–Meyer–Olkin criterion) = 0.75, Bartlett’s test of sphericity *χ*^2^(91) = 702.47, *p* < 0.001; CHR: KMO = 0.58, Bartlett’s test of sphericity *χ*^2^ (91) = 347.76, *p* < 0.001). In the ROP group, four components had eigenvalues over KMO of 1 but the scree plot inflection point justified retaining one major component (Fig. 2b). In the CHR group, five components had eigenvalues over Kaiser’s criterion of 1 but the scree plot inflection point supported to retain three components (Fig. 2c).

As the CHR group presented to be heterogeneous, subjects were subsequently assigned to subgroups according to the highest PCA loading factor following an exploratory approach (Supplementary Material). PCA was not performed in ROD and HC due to low VisDys prevalence rates.

### Associations between VisDys and clinical measures

In ROP, higher VisDys sum score correlated with lower score for functional remission (FROGS-daily life, *τ* = −0.150, *p* = 0.036) and GF Social (*τ* = −0.180, *p* = 0.014) (Table S6).

In CHR, higher VisDys sum scores were associated with lower scores for health-related functional remission (FROGS-Health and Treatment subscale (*τ* = −0.162, *p* = 0.024)), and lower Qol (WHOQOL-physical subscale: *τ* = −0.213, *p* = 0.004; WHOQOL-psychological subscale: *τ* = −0.173, *p* = 0.015). Higher VisDys sum scores were further associated with more severe depression on BDI-II (*τ* = 0.149, *p* = 0.021). Follow-up partial correlation analyses, controlling for BDI-II revealed the extent of depressiveness did not affect the association between VisDys and Qol in CHR, particularly on the physical sublevel (Table S7). Finally, higher VisDys sum scores in CHR were associated with more impaired visuospatial constructability (ROCF-whole, *τ* = −0.162, *p* = 0.027 and ROCF-delayed scores, *τ* = −0.130, *p* = 0.038).

In ROD and HC groups, no relevant correlations were found between VisDys sum scores and any parameters representing functional remission, Qol, depressiveness, or visuospatial constructability (Supplementary Material).

### Predicting VisDys by functional connectivity in ON and FPN

The machine-learning and the original sample showed similar profiles regarding clinical characteristics and symptom expression (Table S8). Specific characteristics of the best models predicting VisDys as revealed by multivariate pattern analyses are presented in Table 2.

**Table 2:** Specification of machine-learning results.

Analysis	BAC (%)	Accuracy (%)	Sensitivity (%)	Specificity (%)	AUC	PPV (%)	NPV (%)	*p* value
ON_ROP	60.17	60.00	80.59	39.74	0.59	56.84	67.50	**0.0001**
ON_CHR	67.38	65.62	44.93	89.83	0.69	83.78	58.24	**0.029**
ON_ROP + CHR	55.89	55.51	44.85	66.92	0.62	59.22	53.13	0.221
ON_ROD	51.57	76.86	13.04	90.09	0.46	21.43	83.33	0.360
FPN_ROP	52.67	52.59	64.18	41.18	0.57	51.81	53.85	0.250
FPN_CHR	63.02	61.72	46.37	79.66	0.67	72.73	55.95	0.100
FPN_ROP + CHR	61.11	60.84	52.94	69.29	0.66	64.86	57.89	**0.006**
FPN_ROD	47.67	76.12	4.35	90.99	0.51	9.09	82.11	0.700
ON-FPN_ROP	56.46	56.29	79.11	33.82	0.59	54.08	62.16	0.222
ON-FPN_CHR	62.77	61.72	49.28	76.27	0.64	70.83	56.25	0.122
ON-FPN_ROP + CHR	59.69	59.32	48.53	70.86	0.64	64.07	56.25	**0.016**
ON-FPN_ROD	41.28	62.68	8.69	73.87	0.37	6.45	79.61	0.956

Significant *p* values are printed in bold type.

*ON* occipital network, *FPN* frontoparietal network, *ON-FPN* occipital and frontoparietal network combined, *CHR* clinical high risk for psychosis, *ROP* recent onset of psychosis, *ROP* *+* *CHR* combined group of ROP and CHR, *ROD* recent onset of depression, *BAC* balanced accuracy, *AUC* area under the curve, *PPV* positive predictive value, *NPV* negative predictive values.

In ROP, the model correctly classified recent VisDys^+^ from VisDys^−^ based on ON connectivity with a BAC of 60.17% (*p* = 0.0001). Sensitivity of the ROP model was high indicating high rates of true predictions of VisDys^+^ based on connectivities in ON whereas specificity was low (Table 2). In CHR, the referring BAC was 67.38% (*p* = 0.029). Here, high specificity indicates high rates of true predictions of VisDys^–^ based on ON connectivity, whereas sensitivity was low (for results regarding the combined ROP + CHR sample see Table 2).

To investigate whether the associations of functional brain alterations with VisDys were similar across disorder groups, we applied the ROP model on the CHR group and vice versa. For both ON and FPN, this was not successful. We also tested the ON-ROP model on the three individual CHR subgroups established by PCA (Fig. 2b, c and Tables S2 and S3). By this exploratory approach, the ON-ROP model correctly identified 9/12 (75.00%) of VisDys^+^ probands in CHR-subgroup 1, 16/19 (84.21%) in CHR-subgroup 2, and 19/30 (63.33%) in CHR-subgroup 3 suggesting that the relationships between VisDys and ON in CHR-subgroup 2 may be similar to ROP.

**Fig. 3:**
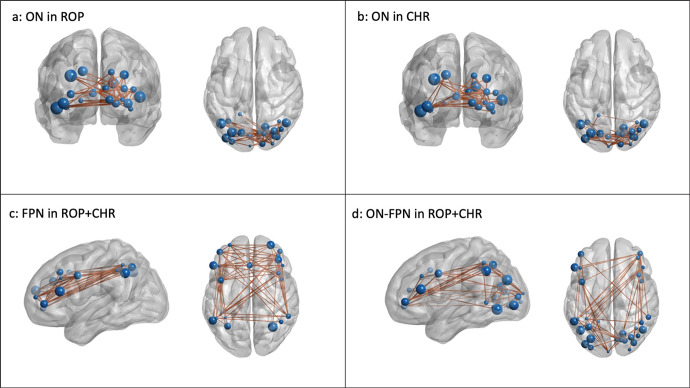
Most reliable intrinsic brain activity connectivities predicting visual dysfunctions. Connectivities (lines) between regions of interest (spheres) derived from resting-state brain activity were identified as classifying the occurrence of visual dysfunctions (VisDys^+^ vs. VisDys^–^) in patients with recent onset psychosis (ROP) and subjects at clinical high risk (CHR). Depicted are the most reliable connectivities, within the occipital network (ON) in ROP (**a**) and CHR groups (**b**). For the combined ROP + CHR group, connectivities within the frontoparietal network (FPN) and the combined ON-FPN are shown in **c**, **d**, respectively. For a list of all cross-validation ratios (CVs) see Tables S9–S12.

The most relevant functional connectivities contributing to the prediction of VisDys are illustrated in Fig. 3 (for a complete list of CVRs see Tables S9–S12).

No significant models predicting VisDys in ROD were established (Table 2). Additionally, the application of significant models revealed in ROP and CHR did not yield any significant predictions in the ROD group.

## Discussion

This study was driven by three major questions:

### Are VisDys specific to the psychosis spectrum at early stages of a mental disorder?

The main findings from this large sample study support the idea of VisDys being specific to the psychosis spectrum already at early stages, i.e., ROP and CHR, while VisDys were reported much less frequent in ROD, and appeared negligible in HC. Thus, our findings add to previous reports on patients with stable states of schizophrenia [5, 45, 46] including similar VisDys sum score levels reported recently from schizophrenia patients in an independent study using the same SPI-A items for VisDys representation [29]. VisDys have been also reported in CHR as they are regarded as basic symptoms mediating susceptibility to psychosis [5, 47–54]. Interestingly, we found higher heterogeneity among individual VisDys phenomena in CHR than ROP, while overall VisDys severity in VisDys^+^ participants was similar in both groups. This constellation of findings suggests that the phenomenon of VisDys should be regarded differently in CHR and ROP as clinical impact and even neurobiological dysfunctions driving the phenomenon of VisDys might differ across psychosis spectrum groups.

### Are VisDys associated with clinical characteristics?

Previously, VisDys have been associated with lower overall functioning, poorer treatment response [2], and higher suicidal ideation in schizophrenia [55]. In our sample, ROP patients were clinically more impaired than CHR participants as reflected by higher PANSS-scores, lower functional remission (FROGS) and overall lower functioning (GF-subscales) in ROP. However, in both ROP and CHR higher VisDys sum scores correlated with lower functional remission, suggesting that VisDys may indicate an impaired ability to take care of oneself and being an independent member of society [32].

Additionally, CHR patients reporting higher VisDys severity showed decreased Qol, an association not seen in ROP patients. This finding suggests greater susceptibility to effects of VisDys on subjective well-being in CHR than ROP. Note, CHR patients generally reported lower levels of Qol compared with ROP. This in turn could be explained by higher suffering from depressive symptoms (BDI-II-scores) in CHR patients, possibly affecting Qol [56]. However, while BDI-II could explain the group differences regarding Qol, the extent of depressiveness did not affect the association between VisDys and Qol in CHR, particularly on the physical subscale.

Additionally, VisDys in CHR were associated to impaired visuospatial constructability (ROCF-whole score), a specific impairment affecting the ability to deconstruct a visual object into set of parts and to then construct a replica from these parts. This finding adds to previous work reporting associations between visual distortions and impaired cognition more generally [5, 57, 58]. The absence of this association in ROP might be explained by both, generally stronger impairment of visuospatial constructability and stronger symptom expression of positive symptoms in ROP than CHR, possibly overshadowing associations with VisDys severity.

Together, the associations of VisDys with lower levels of functioning, Qol and cognitive function in CHR highlight the importance of beneficial interventions in this group [56] with VisDys possibly representing a warning sign for early intervention.

### Is functional intrinsic connectivity within ON and FPN related to VisDys and if so, are there differences in this relationship across the psychosis spectrum?

Only little is known about underlying neurobiological processes leading to or being associated with VisDys. Originating in the retina, visual processing streams pass through the thalamus to the primary visual cortex in occipital cortex (ON). From there visual information is processed along either the dorsal stream to parietal areas, which form part of frontoparietal networks (FPN), or along the ventral stream involving more temporal regions [3]. While the dorsal stream, primarily from magnocellular layers, is generally for initial attentional capture and processing of overall stimulus organization, the ventral stream, primarily from parvocellular layers, processes fine-gained stimulus details and serves for object identification [3]. Note, both streams are highly interconnected [59]. Additionally, bottom-up visual information processing is modulated by top-down control from higher-order frontal networks [23].

In schizophrenia, previous research highlights abnormalities of visual information processing along both visual streams including retinal dysfunctions with a predominance of alterations observed related to the dorsal visual stream [3, 9–13, 60–62]. For instance, basic visual symptoms correlated with rapid visual processing and magnocellular pathway function [63]. From functional brain imaging studies with active task performance, hyper- and hypoactivations in multiple brain areas, including occipital, temporal, parietal, and frontal areas have been reported in schizophrenia, and CHR states [64], which may explain the VisDys phenomenon [65]. For intrinsic, resting-state brain activity, decreased coupling in salience network, dorsostriatal, superior temporal areas have been shown in CHR [65]. Independently from mental states, visual hallucinations in blind probands have been linked to a build-up of resting-state neural activity in early visual systems [66], another study found visual disruptions such as micropsia and macropsia to be linked to alterations in ON and FPN [67].

Our large sample study is among the first to approach the VisDys phenomenon in early psychosis states using a multivariate machine-learning approach based on intrinsic brain connectivity as it allows investigating patterns of multivariable characteristics within brain systems related to VisDys [25]. This approach identified two different predictive ON-models for ROP and CHR, respectively. The ON-model in ROP showed high sensitivity (80.59%) suggesting ON connectivities served well to identify VisDys^+^ participants correctly, however specificity of this model was low. In contrast, the ON-model in CHR showed high specificity (89.83%), but low sensitivity suggesting ON connectivities rather identified VisDys^−^ among CHR participants. Application of the individual ON-ROP model to CHR and vice versa was not successful, underlining the differences between CHR and ROP regarding the relationship between ON and VisDys. This observation adds to the different relations between VisDys and clinical measures in these groups discussed above. However, the exploratory finding that in one of the three CHR subgroups (i.e., component 2, Table S3), the ROP model correctly identified 16 of 19 subjects with VisDys^+^, also suggests that this CHR-subgroup is similar to ROP regarding neurobiological underpinnings of VisDys.

Interestingly, we yielded two additional predictive models for VisDys across ROP + CHR groups, both involving the FPN. The FPN is regarded as mediating sustained attention, including visuospatial attention processed in parietal areas, for cognitively demanding tasks to frontal areas [23, 24]. Thus, while ROP and CHR differed in their relations between VisDys and ON, this constellation of findings suggests common alterations of visual information processing related to VisDys across both groups at higher-order cortical levels. In line with this, resistance to the phenomenon of depth inversion illusions in patients with schizophrenia and CHR-state has been hypothesized to reflect reduced constraints of higher-order top-down control in frontoparietal networks during visual perception [68–70].

In contrast to these findings in ROP and CHR, we did not find any evidence for functional connectivities in ON and FPN being related to VisDys in ROD supporting the notion that disturbances of visual information processing related to intrinsic ON and FPN are indicative of disease mechanisms in psychotic disorders.

### Limitations

First, CHR and ROP differed in age in the machine-learning sample, which was controlled by using age as a covariate in these analyses. Yet, both groups were mainly above the age threshold of 18 years reported to be relevant to the prevalence of VisDys [20, 21], thus, this age difference likely did not affect our results. Second, an independent sample for external ROP-ROP and CHR-CHR validation would have been beneficial and should be subject to future studies. Third, our study did not incorporate psychophysiological experiments to characterize VisDys but relied solely on subjective reports from patients. Fourth, as VisDys are subtle symptoms, future studies should also consider visual hallucinations to capture a broader range of alterations within the visual system. Fifth, despite careful examination, we cannot fully exclude that VisDys reported by patients were caused by organic factors or retinal dysfunctions not evaluated by the PRONIA protocol, which may be the case despite the young age of participants. Sixth, as we could not fully control subjects’ mental activity during resting-state assessment, general group differences in resting-state activities affecting the association with VisDys could be plausible.

To conclude, subtle VisDys should be regarded a frequent phenomenon across the psychosis spectrum, impinging negatively on patients’ current ability to function in several settings of their daily and social life, their Qol and visuospatial abilities. As these parameters are crucial for patients’ future well-being, it is essential to seriously consider VisDys in the clinical setting, especially in CHR.

Our multivariate findings offer novel insights into possible underlying biological mechanisms associated with VisDys, supporting previous models of dysfunctions within occipital and frontoparietal networks implicated in disease mechanisms of psychotic disorders. However, VisDys should be regarded differently in CHR and ROP requiring future studies to decipher characteristics specific to CHR and ROP in the association of VisDys with visual brain system function and dysfunction.

## Supplementary information

Supplementary material VisDys PRONIA rev final

Table S9 ON_ROP_75th percentile

Table S10 ON_CHR_75th percentile

Table S11 FPN_ROP+CHR_75th percentile

Table S12 ON-FPN_ROP+CHR_99th percentile
