# Supplementary material for: The impact of visual dysfunctions in recent-onset psychosis and clinical high-risk state for psychosis
Source: Neuropsychopharmacology. 2022 Aug 18;47(12):2051–60. doi: 10.1038/s41386-022-01385-3 (PMC9556592; doi:10.1038/s41386-022-01385-3)
Supplement: Supplementary file 1 — Supplementary material VisDys PRONIA rev final [file 41386_2022_1385_MOESM1_ESM.docx]

**Supplementary material to “The impact of visual disturbances in recent-onset psychosis and clinical high-risk state for psychosis” by Schwarzer et al.**

**Methods**

1. Assessment of Visual Dysfunctions (VisDys) and computation of the VisDys sum score
   1. Exploratory analyses on characteristics of VisDys on CHR and ROP
2. Assessment of resting-state activity
3. rsfMRI data preprocessing
4. Connectivity matrix computation
5. Machine learning analysis pipeline

**Results**

1. Statistics of group comparisons of clinical measures and VisDys between CHR and ROP
2. Association between VisDys and clinical measures in CHR and ROP
3. Follow-up statistical analyses: Partial correlations of VisDys with QoL in CHR
4. Machine learning sample characteristics
5. Comprehensive results depicting most relevant cross-validation ratios (CVs) from machine learning analyses

**METHODS**

**1. Assessment of VisDys and computation of VisDys sum score:**

For the assessment of VisDys 14 items of the respective section of the Schizophrenia Proneness Instruments for Adults (SPI-A) [1] were selected (Table S1). Next to items that are defined by the mostly brief occurrence of disturbances in the correct visual perception certain characteristics of real objects (e.g., shape, size, color or motionlessness) (F3, O4.1, O4.2, O4.3, O4.4, O4.5, O4.6, O4.8) and items defined by disturbances in general visual perception (i.e., a limited perception of the whole field of vision and a maintenance of what has been seen before) (O4.10, O4.9), this selection included a hypersensitivity to certain correctly perceived visual stimuli (F1), the perception of little objects, similar to floaters, within the eyes that are not assumed to really exist (F2), disturbances in the inner frame of reference of sizes or distances of otherwise correctly perceived objects (O4.7) as well as an involuntary fixation of the attention on correctly perceived visual stimuli of no particular interest (O7). By definition, these VisDys had to be immediately self-recognized as being generated by own mental processes and as a change from the “normal” mental processes (i.e., as a state), and had to occur outside hypnagogic/hypnopompic states and independent of any potential substance use.

As a severity rating, the SPI-A rates the maximum of frequency of items within the past 3 months as: 0= absent (never present); 1= rare (less than once in a month); 2= mild (short periods about once in a month); 3= moderate (several times in a month or weekly); 4= moderately severe (several times in a week); 5= severe (daily, periods of improvement possible); 6=extreme (daily, but not necessarily continuously); 7= has always been present in same severity (trait); 8= definitely present, but severity unknown; 9= symptom definition questionably met.

Since ratings of 7 and 9 are not basic symptoms in the strict sense, these were recoded as 0. Likewise, following the general rule of assuming the lowest possible rating when in doubt, 8 was recoded as 1 (=the lowest possible rating for a basic symptom definitely present). Ratings of 0-6 remained unchanged. Based on the partially recoded ratings, the VisDys sum score (VisDys_sum) was computed. The maximum possible score was therefore 84, while computed sum scores ranged from 0 (minimum) to 66 (maximum). A VisDys_sum of 0 was categorized as VisDys^–^, while a VisDys_sum ≥ 1 was classified as VisDys^+^. Our selection of SPI-A items used in the present study showed high internal consistency over all subjects (n=721), expressed by Cronbachs Alpha=0.78.

**Table S1: SPI-A items used for computation of VisDys_sum**

| Oversensitivity towards light and/or certain visual perception objects (F1) |
| --- |
| Photopsia (F2) |
| Micropsia/macropsia (F3) |
| Near and tele-vision (O4.1) |
| Metamorphopsia (O4.2) |
| Changes in color vision (O4.3) |
| Changed perception of patient's own face (O4.4) |
| Pseudomovements of optic stimuli (O4.5) |
| Diplopia, oblique vision (O4.6) |
| Disturbances of the estimation of distances or sizes (O4.7) |
| Disturbances of the perception of straight lines/contours (O4.8) |
| Maintenance of optic stimuli 'visual echoes' (O4.9) |
| Partial seeing including tubular vision (O4.10) |
| Captivation of attention by details of the visual field (O7) |

- 1. **Exploratory analyses on characteristics of VisDys in CHR and ROP**

The detailed evaluation of the individual items of the SPI-A using PCA revealed different characteristics across the groups as the CHR group presented to be rather heterogeneous with three major components as compared with the ROP group with one major component. (Table S2).

For every subject, scores for each of the three extracted components were computed using the regression method. Subsequently, each subject was assigned to one of the three subgroups characterized by the three components by evaluating the component with the highest score. If the highest component score was negative, the subject was not assigned to any of the subgroups as was the case in 10 CHR subjects. Item loadings for the three components and number of subjects assigned to each of the subgroups can be found in Table S3.

**Table S2: Eigenvalues and percentage of explained variance for ROP and CHR groups**

|  | **ROP** | | **CHR** | |
| --- | --- | --- | --- | --- |
| **Component** | **Eigenvalues** | **% of Variance** | **Eigenvalues** | **% of Variance** |
| **1** | **4.68** | **33.44** | **2.60** | **18.56** |
| **2** | 1.50 | 10.74 | **2.01** | **14.38** |
| **3** | 1.27 | 9.04 | **1.51** | **10.81** |
| **4** | 1.05 | 7.50 | 1.14 | 8.12 |
| **5** | 0.93 | 6.64 | 1.08 | 7.73 |
| **6** | 0.83 | 5.92 | 0.92 | 6.60 |
| **7** | 0.78 | 5.57 | 0.88 | 6.30 |
| **8** | 0.68 | 4.87 | 0.85 | 6.09 |
| **9** | 0.63 | 4.52 | 0.76 | 5.42 |
| **10** | 0.47 | 3.35 | 0.59 | 4.21 |
| **11** | 0.45 | 3.24 | 0.54 | 3.85 |
| **12** | 0.30 | 2.13 | 0.46 | 3.26 |
| **13** | 0.25 | 1.77 | 0.38 | 2.69 |
| **14** | 0.18 | 1.26 | 0.28 | 2.00 |

**Table S3: Rotated loadings for CHR group based on the three component solution**

| **SPIA Item** | **Component 1** | **Component 2** | **Component 3** |
| --- | --- | --- | --- |
| Oversensitivity to light/visual objects | 0.31 | 0.23 | **0.42** |
| Photopsia | 0.06 | 0.04 | **0.71** |
| Micropsia/macropsia | -0.07 | **0.71** | 0.15 |
| Near and tele-vision | 0.09 | **0.58** | -0.09 |
| Metamorphopsia | 0.11 | 0.10 | -0.09 |
| Changes colour vision | **0.54** | -0.09 | **0.55** |
| Changed perception of own face | 0.06 | **0.58** | 0.35 |
| Pseudomovements optic stimuli | 0.14 | **0.73** | -0.15 |
| Diplopia | **0.84** | 0.02 | -0.04 |
| Estimation of distances/sizes | -0.10 | **0.50** | **0.50** |
| Perception of straight lines/contours | **0.78** | 0.09 | -0.08 |
| Maintenance of optic stimuli | **0.67** | 0.03 | -0.02 |
| Tubular vision | -0.15 | 0.10 | 0.37 |
| Captivation by visual details | -0.11 | -0.22 | **0.48** |
| **n_subjects_ assigned** | **13** | **22** | **35** |

Please note that loadings ≥0.40 are displayed in bold. There are 143 CHR participants in total. n=63 CHR participants had no VisDys and could therefore not be assigned to any group. In n=10 participants component scores were negative so that participants could not be clearly allocated to one of the three subgroups. Therefore, n=70 CHR participants could be included in the analyses.

**2. Assessment of resting-state activity**

MRI data were acquired across all seven sites. To facilitate the evaluation of real-world generalizability, a minimal MRI harmonization protocol was implemented. As part of this approach [2,3], between-site reliability was conducted by sending HC participants to each study site where they were examined with the same functional and structural MRI sequences as clinical participants. While acquiring brain resting-state activity, subjects were instructed to keep their eyes open and not to think about anything. Using their resting-state functional MRI data, we computed a generalization theory map based on the region-of-interest (ROI)-by-ROI correlation matrices (see [4] for the method applied to structural imaging data). The MRI sequence parameters used at each PRONIA site are detailed in [4].

**3. Preprocessing of resting-state MRI**

RsfMRI preprocessing was previously described by Haas et.al. [5] and divided into two processes based on the procedure described by Patel et al. [6]. Core processing was applied using the Statistical Parametric Mapping, version 12 (SPM12) [7] and consisted of initially discarding the first 8 volumes, while the remaining 192 images were slice-time corrected. For head-motion correction, the remaining 192 images were subsequently unwarped and realigned to the first volume. To yield the estimated time course of head motion, translations in each direction and rotations in angular motion around each axis for each volume were estimated. For each subject, the framewise displacement (FD) was calculated, while the FD for the first volume of a run was set to 0. Subjects with a mean FD >0.5 mm in more than 38.5% of volumes were excluded [8]. Next, the affine image coregistration to structural images was applied*.* Afterwards, structural images were resliced using 4^th^-degree B-Spline interpolation. In order to normalize all coregistered images to MNI space, using the SPM12-s population to International Consortium for Brain Mapping 152 registration procedure [9], the standard CAT 12 template was converted from DARTEL space to MNI space. The resulting image was used as the deformation field for normalization. Using an image calculator procedure in SPM 12 masks for grey matter (GM; threshold= 0.20), white matter (WM; threshold: 0.20), and cerebrospinal fluid (CSD; threshold= 0.50) were created. Afterwards, based on Satterthwaite et al [10] Friston 24 motion parameters were derived. These consist of 6 motion parameters, 6 temporal derivatives, 6 quartic terms, and 6 quartic expressions of the derivatives of motion estimates. The variance from WM and CSF was regressed out and therefore individual signal estimates were generated. To limit space functional volumes were masked with the GM mask. For spatial smoothing, a Gaussian kernel of 6 mm full width at half-maximum was used.

Denoising was performed using time series despiking (Wavelet Despike) with the BrainWavelet Toolbox (http://www.brainwavelet.org/) [6]. Using the Resting-State fMRI Data Analysis Toolkit (REST version 1.8; http://www.restfmri.net) [11] confound signal regression of the Friston 24 motion parameters, and residuals of WM and CSF was applied. The images underwent background filtering. In order to reduce the effects of low-frequency drift and high-frequency noise, temporal band-pass filtering (0.01-0.08 Hz) was done.

**4. Connectivity matrix computation**

Subsequently to the rsfMRI preprocessing, functional connectivity selection was based on extensive work done by Dosenbach et al. [12]. Dosenbach and colleagues generated blood-oxygen level dependent (BOLD) time courses for 160 regions of interest (ROIs) covering most of the brain derived from on a series of meta-analyses of fMRI studies. Each ROI is assigned to one of six subnetworks: occipital, frontoparietal, cerebellar, the cingulo-opercular, the default mode and the sensorimotor network. From all these 160 ROIs all possible interregional correlations i.e. functional connections (n=12,720) were extracted. Based on our a priori hypothesis we focused on ROIs associated with occipital (n=22 ROIs) and frontoparietal networks (n=21 ROIs) (Table S4). For our machine-learning analysis we used pairwise ROIxROI functional connectivities within these networks, i.e. 231 resting-state-functional-connectivity (rsFC) for ON and 190 rsFC for FPN.

The application of the Dosenbach atlas was performed in the following manner*:* After using the MarsBaR Toolbox version 0.42 [13] to extract the mean signal from 10mm spheres at each ROI, Pearson’s correlation of average time series between pairwise ROI’s was calculated. This was performed within Matlab R2015 using an in-house-script. This eventually resulted in the 12720 rsFC for every participant in each study group.

**Table S4: ROI’s affiliated with the occipital (ON) and frontoparietal network (FPN) according to Dosenbach et al. [12]**

|  | occipital network | | |  | |  |  |  | frontoparietal network | | | |  |  |
| --- | --- | --- | --- | --- | --- | --- | --- | --- | --- | --- | --- | --- | --- | --- |
| label | | x | y | | z | |  | label | | x | y | z | | |
| occipital | | -18 | -50 | | 1 | |  | aPFC | | 29 | 57 | 18 | | |
| occipital | | -34 | -60 | | -5 | |  | aPFC | | -29 | 57 | 10 | | |
| occipital | | 36 | -60 | | -8 | |  | vent aPFC | | 42 | 48 | -3 | | |
| temporal | | 46 | -62 | | 5 | |  | vent aPFC | | -43 | 47 | 2 | | |
| occipital | | -44 | -63 | | -7 | |  | vlPFC | | 39 | 42 | 16 | | |
| occipital | | 19 | -66 | | -1 | |  | dlPFC | | 40 | 36 | 29 | | |
| occipital | | 17 | -68 | | 20 | |  | ACC | | -1 | 28 | 40 | | |
| occipital | | 39 | -71 | | 13 | |  | dlPFC | | 46 | 28 | 31 | | |
| occipital | | 29 | -73 | | 29 | |  | vPFC | | -52 | 28 | 17 | | |
| occipital | | -29 | -75 | | 28 | |  | dlPFC | | -44 | 27 | 33 | | |
| occipital | | -16 | -76 | | 33 | |  | dFC | | 40 | 17 | 40 | | |
| occipital | | 9 | -76 | | 14 | |  | dFC | | 44 | 8 | 34 | | |
| occipital | | 15 | -77 | | 32 | |  | dFC | | -42 | 7 | 36 | | |
| occipital | | 20 | -78 | | -2 | |  | IPL | | -41 | -40 | 42 | | |
| post occipital | | -5 | -80 | | 9 | |  | IPL | | 54 | -44 | 43 | | |
| post occipital | | 29 | -81 | | 14 | |  | post parietal | | -35 | -46 | 48 | | |
| post occipital | | 33 | -81 | | -2 | |  | IPL | | -48 | -47 | 49 | | |
| post occipital | | -37 | -83 | | -2 | |  | IPL | | -53 | -50 | 39 | | |
| post occipital | | -29 | -88 | | 8 | |  | IPL | | 44 | -52 | 47 | | |
| post occipital | | 13 | -91 | | 2 | |  | IPS | | -32 | -58 | 46 | | |
| post occipital | | 27 | -91 | | 2 | |  | IPS | | 32 | -59 | 41 | | |
| post occipital | | -4 | -94 | | 12 | |  |  | |  |  |  | | |

ACC= anterior cingulate cortex; aPFC=anterior prefrontal cortex; dFC= dorsal frontal cortex ; dlFC= dorsolateral frontal cortex; IPL= inferior parietal lobule; IPS= intraparietal sulcus; temporal= temporal lobe; occipital= occipital lobe; post occipital= posterior occipital lobe; post parietal= posterior parietal lobe; vent aPFC= ventral anterior prefrontal cortex;

**5. Machine learning analysis pipeline**

A sufficiently large sample was entered into the machine learning pipeline [14]: 135 ROP, 128 CHR and 134 ROD participants. The machine learning platform NeuroMiner [15], version 1.0, was used to set up the machine learning analysis pipeline.

First, in order to prepare the data, we have applied the following preprocessing steps: Within the cross-validation framework, connectivity data were ranked using the previously generated between-site voxel reliability map (gmask) [4]. Consistent with previous works [4,20,21], PCA was applied to reduce dimensionality using a limited number of principal components [PC, 11-19] to keep the generalization error low and reduce the risk of overfitting [22]. The PC scores were subsequently scaled from -1 to 1 [4,23]. Afterwards a sequential forward feature selection procedure [24,25] was applied. Hereby, variable sets are evaluated with a wrapper algorithm and based on the performance, the predictive value, variables are selected. To avoid overfitting, the wrapper algorithm was stopped when 20% of the variables from the feature pool were included into the optimally predictive variable subspace [24,25].

Second, regarding the machine parameters, we have used a support-vector machine with a gaussian RBF kernel (kernel width γ=[2^-3^ – 2^3^]). The C (misclassification cost) parameter ranged from 2^-3^ to 2^4^. In order to optimize the search for an optimal class weight we used an additional hyperparameter that multiplies the inverse ratio by a scaling factor (sc=[1 - 2^4^]) prior to multiplication with the C [23].

Third, in order to evaluate the results, we have used repeated nested cross validation consisting of two cross validation cycles, an outer CV cycle (CV2) with 7 folds and an inner CV cycle (CV1) with 6 folds. Generally, the models are trained in the inner CV1 scheme and subsequently the best performing models are applied to the outer CV2 scheme. The separation of the two cycles mitigates against overfitting [18]. Since our data was assessed at 7 different sites, a leave-one-site-out CV was used in order to generate geographical generalization [4,19]. With regards to the repeated-nested-CV this means, that in each partition one site is held out. Models are trained in the remaining six sites (CV1) and the best performing model (based on highest balanced accuracy) is then applied to the left-out site (CV2). This procedure was then repeated for a total of 7 times. Additionally, Cross-Validation Ratios (CVR = mean(w) / standard error (w)) [4,21] were extracted to represent the reliability of each connection. The w in the CVR formula stands for the normalized individual weights from the SVM models generated in the CV scheme, while normalization is created using the Euclidian norm of w, which is defined as s=w/||w||_2_[4,21]_._ CV-Ratios were extracted at the 75^th^ Percentile for ON and FPN and the 99^th^ percentile for ON-FPN. For ON and FPN the 75^th^ percentile was chosen to generate clearness of the visualization. In addition, the 75^th^ percentile was extracted as opposed to a higher percentile, for these analyses comprise a rather small number of connections. A positive CVR is more characteristic for the presence of VisDys (VisDys^+^), whereas a negative CVR indicated a greater characteristic for the absence of VisDys (VisDys^–^) within the last three month.

Fourth, permutation analysis was performed in order to obtain statistical significance of the prediction [26–28]. A null distribution of out-of-training classification performance was created. The null distribution generation was performed as follows. 1000 random permutations were performed for the outcome labels (VisDys^+^; VisDys^–^) in each intrinsic brain network for each study group. All SVM models were retrained in the leave-one-site out repeated nested CV with feature subsets acquired from the observed-label analyses. Prediction of random models were collected for each permutation into a permuted prediction for each outer cycle participant. Finally, the number of events when the permuted out-of-training accuracy was higher or equal to the observed accuracy was divided by the number of total permutations performed. The α-level of the model was set at α=0.05 (see Antonucci et. al for a detailed description [21]).

**RESULTS**

**6. Statistics of group comparisons of clinical measures and VisDys between CHR and ROP**

**Table S*5*: Group Comparisons of clinical parameters between CHR and ROP**

| Clinical measures | p-value, T, df |
| --- | --- |
| VisDys sum score | p=0.490; T=-0.69; df=288 |
| PANSS_pos | p=**2.45e-28,** T=-12.76; df=223 |
| PANSS_neg | p=**2,13e-7,** T=-5.32; df=275 |
| PANSS_gen | p=**1,48e-12,** T=-7.44, df=260 |
| BDI II | p=**0.011,** T=2.55, df=264 |
| WHOQOL_physical | p**=-0.038**, T=-2.08, df=257 |
| WHOQOL_psychological | p=**3.80e-5,** T=-4.19, df= 262 |
| WHOQOL_social | p=0.485, T=-0.70, df=256 |
| WHOQOL_environmental | p=0.518, T=0.65, df=258 |
| FROGS_DailyLife_Subscale | p=**3.00e-6**, T=4.75, df=289 |
| FROGS_Activities_Subscale | p=**3.80e-5** T=4.18, df=277 |
| FROGS_Relationships_Subscale | p=**9.60e-5**, T=4.00, df=289 |
| FROGS_Health and Treatment_Subscale | p=**2.25e-4**, T=3.74, df=288 |
| ROCF_Score_whole | p=**0.027**, T=2.22, df=245 |
| ROCF_whole_Immediate | p=**8.10e-5**, T=4.00, df=287 |
| ROCF_whole_Delayed | p=**0.004**, T=3.18, df=287 |
| GF_Role | p=**4.27e-8**, T=5.32, df=292 |
| GF_Social | p=**2.09e-7**, T=5.63, df=292 |

Displayed are the differences between CHR and ROP with regards to the parameters shown. P-values significant at an α=0.05 are printed bold.

**7. Associations between VisDys and clinical measures in CHR, ROP and ROD**

**Table S6: Associations between VisDys and clinical measures across groups**

|  | **VisDys in ROP** | **VisDys in CHR** | **VisDys in ROD** |
| --- | --- | --- | --- |
| PANSS_pos | τ=0.076, p=0.465 | τ= 0.119, p=0.102 | τ= 0.225, **p=0.009*** |
| PANSS_neg | τ=0.088, p=0.465 | τ=0.009, p=0.514 | τ=0.070, p=0.604 |
| PANSS_gen | τ=0.074, p=0.465 | τ=0.041, p=0.514 | τ=-0.030, p=0.651 |
| BDI II | τ=0.049, p=0.451 | τ=0.149, **p=0.021** | τ=0.087, p=0.204 |
| WHOQOL_physical | τ=-0.122, p=0.140 | τ=-0.210, **p=0.004** | τ=-0.141, p=0.176 |
| WHOQOL_psychological | τ=-0.052, p=0.218 | τ=-0.170, **p=0.015** | τ=-0.027, p=1.000 |
| WHOQOL_social | τ=-0.113, p=0.153 | τ=-0.025, p=0.357 | τ=-0.006, p=1.000 |
| WHOQOL_environmental | τ=-0.083, p=0.208 | τ=-0.087, p=0.192 | τ=-0.014, p=1.000 |
| FROGS_DailyLife_Subscale | τ=-0.150, **p=0.036** | τ=-0.130, p=0.072 | τ=0.069, p=0.924 |
| FROGS_Activities_Subscale | τ=-0.122, p=0.069 | τ=-0.102, p=0.110 | τ=-0.005, p=1.000 |
| FROGS_Relationships_Subscale | τ=-0.123, p=0.069 | τ=-0.024, p=0.352 | τ=-0.008, p=1.000 |
| FROGS_Health and Treatment_Subscale | τ=-0.127, p=0.069 | τ=-0.162, **p=0.024** | τ=0.091, p=0.756 |
| ROCF_Score_whole | τ=-0.056, p=0.597 | τ=-0.160, **p=0.027** | τ=0.087, p=0.672 |
| ROCF_whole_Immediate | τ=-0.088 p=0.597 | τ=-0.096, p=0.064 | τ=-0.096, p=0.672 |
| ROCF_whole_Delayed | τ=-0.074, p=0.597 | τ=-0.130, **p=0.038** | τ=0.082, p=0.672 |
| GF_Role | τ=-0.090, p=0.171 | τ=-0.004, p=1.000 | τ=-0.027, p=0.796 |
| GF_Social | τ=-0.180, **p=0.014** | τ=-0.020, p=1.000 | τ=-0.061, p=0.796 |

Depicted are Kendall’s tau values with corrected p-values of the correlations between the clinical parameter and the VisDys sum score. Corrected p-values significant at an α=0.05 are printed bold.

*Note, mean PANSS_pos in ROD was 7.99 making this correlation clinically irrelevant (Table 1).

**8. Follow-up statistical analysis: Partial correlations of VisDys with QoL in CHR**

As in CHR in contrast to ROP, higher VisDys sum scores were associated with lower levels of quality of life, indicated by WHOQOL_physical and WHOQOL_psychological, we conducted further analyses. Note, that CHR scored significantly lower on WHOQOL subscales (p<0.01) than ROP patients. We hypothesized that this could be due to higher rates of depressiveness in the CHR group, measured by the BDI-II (p<0.05). In order to ensure that the correlation between VisDys sum score and WHOQOL physical and psychological subscale observed in CHR was not solely driven by the BDI-II we conducted a partial correlation, controlling for BDI-II. The results can be seen in Table S7. Our results show that on the physical subscale of WHOQOL the correlation with the visual disturbances withstands controlling for BDI-II.

**Table S7: Partial correlation results of VisDys with QoL in CHR implementing BDI-II as the covariate**

|  | WHOQOL physical | WHOQOL psychological |
| --- | --- | --- |
| VisDys sum score | -.218 (p=0.016) | -.108 (p=0.237) |

Displayed are the correlation coefficients and the p-values of the partial correlation. BDI-II was controlled for as the covariate.

**9. Machine learning sample characteristics**

**Table S8: Machine learning Sample- Group comparisons (see also Table 1 in main manuscript)**

|  | **ROD** | **CHR** | **ROP** | *Statistics* |
| --- | --- | --- | --- | --- |
| *N** | 134 | 128 | 135 |  |
| *Age (SD)* | 29.24 (6.16) ^c^ | 26.83 (4.96) ^b,d^ | 28.53 (5.59)^c^ | *F(2,394)=6.38, p=0.002* |
| *Sex (m:w)* | 60:74 ) ^d^ | 68:60 | 80:55) ^b^ | *X^2^(2, N=397)=5.70, p=0.058* |
| *VisDys Prevalence (+/-)* | 17.16% (111/23)) ^c,d^ | 53.90% (69/59) | 49.60% (67/68)) | *X^2^(2, N=397)=44.62, p<0.001* |
| *VisDys sum score (SD)* | 0.59 (1.83) ^c,d^ | 3.71 (5.57) ^b^ | 4.32 (8.78) ^b^ | *F(2,393)=14.13, p<0.001* |
| *WHOQOL physical (SD)* | 12.83 (2.70) | 13.05 (2.37) | 13.65 (2.61) | *F(2,351)=3.21, p=0.041* |
| *WHOQOL psychological (SD)* | 10.32 (2.62) ^d^ | 10.60 (3.03)^d^ | 12.19 (3.15) ^b,c^ | *F(2,351)=13.98, p<0.001* |
| *WHOQOL social (SD)* | 12.33 (3.51) | 12.31 (3.73) | 12.82 (3.59) | *F(2,345)=0.75, p=0.474* |
| *WHOQOL environmental (SD)* | 14.40 (2.57) | 14.17 (2.26) | 14.01 (2.58) | *F(2,348)=0-74, p=0.478* |
| *FROGS DailyLife (SD)* | 20.27 (4.25) ^d^ | 19.40 (4.56) ^d^ | 16.95 (4.69) ^b,c^ | *F(2,391)=19.51, p<0.001* |
| *FROGS Activities (SD)* | 9.26 (3.52) ^d^ | 9.07 (3.53) ^d^ | 7.42 (2.97) ^b,c^ | *F(2,391)=12.23, p<0.001* |
| *FROGS Relationships (SD)* | 18.11 (4.49) ^c,d^ | 16.90 (4.30) ^b,d^ | 15.13 (4.20) ^b,c^ | *F(2,391)=15.94, p<0.001* |
| *FROGS Health and Treatment (SD)* | 11.95 (2.76) ^c,d^ | 11.25 (2.57) ^b,d^ | 10.19 (2.75) ^b,c^ | *F(2,390)=14.46, p<0.001* |
| *PANSS pos (SD)* | 7.65 (1.19) ^c,d^ | 10.18 (2.95)^b,d^ | 17.48 (6.29)^b,c^ | *F(2,391)=207.27, p<0.001* |
| *PANSS neg (SD)* | 12.37 (4.82) ^d^ | 12.26 (5.76)^d^ | 16.35 (7.91) ^b,c^ | *F(2,391)=18.10, p<0.001* |
| *PANSS gen (SD)* | 27.33 (6.72) ^d^ | 27.65 (6.81)^d^ | 35.66 (10.65) ^b,c^ | *F(2,387)=42.45, p<0.001* |
| *ROCF whole (SD)* | 33.89 (2.85) | 34.26 (2.58) ^d^ | 33.33 (3.96) ^c^ | *F(2,377)=2.78, p=0.063* |
| *ROCF immediate (SD)* | 23.64 (6.09) ^d^ | 22.78 (6.63) ^d^ | 19.73 (6.93) ^b,c^ | *F(2,375)=12.60, p<0.0001* |
| *ROCF Delayed (SD)* | 23.53 (6.19) ^d^ | 22.19 (6.48) ^d^ | 19.87 (6.83) ^b,c^ | *F(2,379)=10.52, p<0.001* |
| *BDI II (SD)* | 26.34 (13.77) ^d^ | 25.79 (12.59)^d^ | 22.00 (12.45) ^b,c^ | *F(2,355)=4.01, p=0.019* |
| *GF Role (SD)* | 6.29 (1.64) ^d^ | 6.21 (1.40) ^d^ | 5.18 (1.70) ^b,c^ | *F(2,393)=20.53, p<0.001* |
| *GF_Social (SD)* | 6.49 (1.32) ^d^ | 6.45 (1.26) ^d^ | 5.63 (1.50) ^b,c^ | *F(2,393)=16.75, p<0.001* |

**10. For comprehensive results depicting most relevant cross-validation ratios (CVs) from machine learning analyses see separate tables in Excel format**

Table S9: Cross-validation ratios (CVs) of features in the 75^th^ percentile within the occipital network (ON) in ROP

Table S10: Cross-validation ratios (CVs) of features in the 75^th^ percentile within the occipital network (ON) in CHR

Table S11: Cross-validation ratios (CVs) of features for the 75^th^ percentile within the frontoparietal network (FPN) for ROP+CHR

Table S12: Cross-validation ratios (CVs) of features for the 99^th^ percentile within the combined ON-FPN network for ROP+CHR

**References**

1. Schultze-Lutter F, Addington J, Ruhrmann S, Klosterkötter J. The Schizophrenia Proneness Instrument, Adult version (SPI-A). Giovanni Fioriti Editore; 2007.

2. Mushquash C, O’Connor BP. SPSS and SAS programs for generalizability theory analyses. Behav Res Methods. 2006;38:542–547.

3. Brennan RL. Generalizability theory and classical test theory. Appl Meas Educ. 2011;24:1–21.

4. Koutsouleris N, Kambeitz-Ilankovic L, Ruhrmann S, Rosen M, Ruef A, Dwyer DB, et al. Prediction Models of Functional Outcomes for Individuals in the Clinical High-Risk State for Psychosis or with Recent-Onset Depression: A Multimodal, Multisite Machine Learning Analysis. JAMA Psychiatry. 2018;75:1156–1172.

5. Haas SS, Antonucci LA, Wenzel J, Ruef A, Biagianti B, Paolini M, et al. A multivariate neuromonitoring approach to neuroplasticity-based computerized cognitive training in recent onset psychosis. Neuropsychopharmacology. 2020:1–8.

6. Patel AX, Kundu P, Rubinov M, Jones PS, Vértes PE, Ersche KD, et al. A wavelet method for modeling and despiking motion artifacts from resting-state fMRI time series. Neuroimage. 2014;95:287–304.

7. Functional Imaging Laboratory. SPM12 Software - Statistical Parametric Mapping. 2014.

8. Power JD, Mitra A, Laumann TO, Snyder AZ, Schlaggar BL, Petersen SE. Methods to detect, characterize, and remove motion artifact in resting state fMRI. Neuroimage. 2014;84:320–341.

9. McConnell Brain Imaging Centre - McGill University. ICBM 152. 2009.

10. Satterthwaite TD, Elliott MA, Gerraty RT, Ruparel K, Loughead J, Calkins ME, et al. An improved framework for confound regression and filtering for control of motion artifact in the preprocessing of resting-state functional connectivity data. Neuroimage. 2013;64:240–256.

11. Song X-W, Dong Z-Y, Long X-Y, Li S-F, Zuo X-N, Zhu C-Z, et al. REST: a toolkit for resting-state functional magnetic resonance imaging data processing. PLoS One. 2011;6:e25031.

12. Dosenbach NUF, Nardos B, Cohen AL, Fair DA, Power JD, Church JA, et al. Prediction of individual brain maturity using fMRI. Science (80- ). 2010;329:1358–1361.

13. Brett M, Anton J-L, Valabregue R, Poline J-B. Region of interest analysis using an SPM toolbox2016.

14. Flint C, Cearns M, Opel N, Redlich R, Mehler DMA, Emden D, et al. Systematic misestimation of machine learning performance in neuroimaging studies of depression. Neuropsychopharmacology. 2021;46:1510–1517.

15. Koutsouleris N. Neurominer. http://proniapredictors.eu/neurominer/index.html. Accessed 20 January 2021.

16. Orban P, Desseilles M, Mendrek A, Bourque J, Bellec P, Stip E. Altered brain connectivity in patients with schizophrenia is consistent across cognitive contexts. J Psychiatry Neurosci. 2017;42:17–26.

17. Kapur S, Phillips AG, Insel TR. Why has it taken so long for biological psychiatry to develop clinical tests and what to do about it. Mol Psychiatry. 2012;17:1174–1179.

18. Wei Y, Chang M, Womer FY, Zhou Q, Yin Z, Wei S, et al. Local functional connectivity alterations in schizophrenia, bipolar disorder, and major depressive disorder. J Affect Disord. 2018;236:266–273.

19. Wu X, Zeng L-L, Shen H, Yuan L, Qin J, Zhang P, et al. Functional network connectivity alterations in schizophrenia and depression. Psychiatry Res Neuroimaging. 2017;263:113–120.

20. Koutsouleris N, Riecher-Rössler A, Meisenzahl EM, Smieskova R, Studerus E, Kambeitz-Ilankovic L, et al. Detecting the Psychosis Prodrome Across High-Risk Populations Using Neuroanatomical Biomarkers. Schizophr Bull. 2014;41:471–482.

21. Antonucci LA, Penzel N, Pergola G, Kambeitz-Ilankovic L, Dwyer D, Kambeitz J, et al. Multivariate classification of schizophrenia and its familial risk based on load-dependent attentional control brain functional connectivity. Neuropsychopharmacology. 2020;45:613–621.

22. Hansen LK, Larsen J, Nielsen FÅ, Strother SC, Rostrup E, Savoy R, et al. Generalizable patterns in neuroimaging: How many principal components? Neuroimage. 1999;9:534–544.

23. Dwyer DB. NeuroMiner Manual. 2017.

24. Dwyer DB, Falkai P, Koutsouleris N. Machine Learning Approaches for Clinical Psychology and Psychiatry. Annu Rev Clin Psychol. 2018;14:91–118.

25. Saeys Y, Inza I, Larrañaga P. A review of feature selection techniques in bioinformatics. Bioinformatics. 2007;23:2507–2517.

26. Lessov-Schlaggar CN, Rubin JB, Schlaggar BL. The Fallacy of Univariate Solutions to Complex Systems Problems. Front Neurosci. 2016;10:267.

27. Golland P, Fischl B. Permutation tests for classification: Towards statistical significance in image-based studies. In: Taylor C, Noble JA, editors. Lect. Notes Comput. Sci. (including Subser. Lect. Notes Artif. Intell. Lect. Notes Bioinformatics), vol. 2732, Springer, Berlin, Heidelberg; 2003. p. 330–341.

28. Koutsouleris N, Kahn RS, Chekroud AM, Leucht S, Falkai P, Wobrock T, et al. Multisite prediction of 4-week and 52-week treatment outcomes in patients with first-episode psychosis: a machine learning approach. The Lancet Psychiatry. 2016;3:935–946.
